# Tips for fundraising

**Published:** 2013

**Authors:** Claire Walker

**Affiliations:** Funding Advisor: VISION 2020 LINKS Programme, London School of Hygiene & Tropical Medicine, London, UK. **claire.walker@lshtm.ac.uk**

**Figure:**
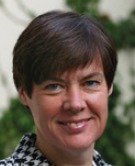
Claire Walker

## 1 Make the case for support

The key is to answer the question ‘What difference will it make?’ in terms that ordinary people (non-clinicians) can understand, and with which they can identify. Always relate your project or programme to people: what are their lives like now and how will your project improve things? How many people will benefit?

## 2 Decide who to ask

Start with people you know already: existing and past donors, patients, Ministry of Health, non-governmental organisations (NGOs). Follow up with your suppliers – i.e. drugs and equipment companies – they may support you financially or give gifts ‘in kind’. Find out which are the active community groups in your area, e.g. Lions, Rotary, or churches. Find out who funds other projects in your area – annual reports of charities and universities often list donors. Ask around, network, and use the internet to find sources of funding.

## 3 How to ask for funding

- You would be surprised how many people you know who could intervene on your behalf. Who might be able to introduce you to a potential donor? Make your case to them and get them on your side so they can be an ambassador for you.
- When you make contact with a potential donor, invite them to meet you. Say something like: ‘I have an exciting new project, I need your advice, would you come and see me?’ If you were introduced by one of your ‘ambassadors’, make sure they can be there when the donor visits.
- ‘Sell’ the project to the donor before you ask them for money. Make the case for support. Once the donor is on your side, tell them what you need.
- Always ask face-to-face. Don't let someone go until you have asked them for precisely what you need – then wait for their reaction.

It will take time at first, but if you can get a handful of people ‘on board’, give them feedback, and keep them involved, you should be able to secure funding for a number of years.

